# Relationship between Occupational Stress and Work-related Musculoskeletal Disorders in Korean Male Firefighters

**DOI:** 10.1186/2052-4374-25-9

**Published:** 2013-07-04

**Authors:** Min Gi Kim, Kyoo-Sang Kim, Jae-Hong Ryoo, Seung-Won Yoo

**Affiliations:** 1 Department of Occupational & Environmental Medicine, Dongguk University, Gyeongju, South Korea; 2 Center for Occupational Disease Research, Occupational Safety and Health Research Institute, Korea Occupational Safety and Health Agency, Incheon, South Korea; 3 Department of Preventive Medicine, School of Medicine, Kyung Hee University, Seoul, South Korea

**Keywords:** Work-related musculoskeletal disorder, Firefighter, Job stress

## Abstract

**Objectives:**

A growing body of literature has documented that job stress is associated with the development of work-related musculoskeletal disorders (WMSDs). However, the association of WMSDs with job stress has not yet been fully studied in Korean male firefighters. The purpose of this study was to determine the status of WMSDs in almost all Korean male firefighters and to clarify the effect of job stress on the occurrence of WMSDs.

**Methods:**

The study design was cross-sectional, and 21,466 firefighters were recruited. The study design included a structured questionnaire to assess general characteristics, the Korean Occupational Stress Scale (optional KOSS-26), Center for Epidemiologic Studies-Depression Scale (CES-D), and WMSDs. The chi-square test, and univariate and multivariate logistic regression analyses were used to look for a correlation between general characteristics and job stress, and the occurrence of WMSD.

**Results:**

Back pain is the most common WMSD. Among the job stress subgroup, physical environment, job demands, organizational system, occupational climate, lack of reward and job insecurity were related to the occurrence of WMSDs. However, insufficient job control and interpersonal conflict were not related to the occurrence of WMSDs.

**Conclusion:**

Job stress was related to the occurrence of WMSDs in Korean male firefighters. To reduce the occurrence of WMSDs, a job stress management program may be required.

## Introduction

In South Korea, firefighters are public employees who prevent and suppress fire, perform emergency rescues, and respond to disasters in a recovery role. They are also exposed to physical dangers including heat, noise, poisonous gases, smoke, carbon monoxide, and diesel fumes [1]. Moreover, they are exposed to traumatic accidents and mental diseases [2,3]. The rate of post-traumatic stress disorder [2-5], mortality by cardiovascular disease [6], musculoskeletal disease [7], and oxidative DNA damage [8] are higher in firefighters than general population. The relationship between these diseases and job stress and shift work [9], overtime work, a heavy workload [10,11], discord at work, low social support, role conflicts, and low self-esteem [12] have been studied in firefighters. In Korean firefighters the turnover decision factor [13], job satisfaction [14], occupational climate [15], conduct of business [16], and safety-related accident factors [17] have been studied.

Because of heavy equipment, fire-fighting requires excessive energy and awkward postures, so work-related musculoskeletal disorders(WMSDs) can easily develop. According to United States statistics from 2007, musculoskeletal system diseases like sprains of the muscle or ligament were over 40% of the problems that developed in fire fighting [18]. From 2007, work-related accidents were reported for 279 people among 30,630 Korean firefighters. However, there is no information about WMSDs in Korea [18].

Only one study has addressed the association between WMSDs and job stress in Korean firefighters [19], but it did not adequately confirm the relationship between job stress and WMSDs. Also, the study population was small. The purpose of this study was to determine the status of WMSDs in almost all male Korean firefighters and to understand the effect of job stress on the occurrence of WMSDs.

## Materials and Methods

### Study subjects

Between early July 2007 and the end of November 2007, structured questionnaires were distributed to 30,601 Korean firefighters. In total, 25,610 (83.6%) of the firefighters responded to the questionnaire. The 1292 female fighters composed only a small portion (4.9%) of the total, so we excluded female firefighter cases. Among those who respond to the questionnaire, only 21,466 (83.8%) firefighters completed the questionnaire, so they were selected as the final subjects.

### The method of study and definition

The structured questionnaires had 4 sections; general characteristic factors; work-related job stress factors, depression factors, and WMSDs. The general characteristic factors comprised age, marital status, drinking habits, smoking habits, sports-playing habits, level of education, and work department (Firefighting, Rescue, Emergency Medical Services, or Other). The level of education was defined as at least 16 years or less than that, based on the time required for university graduation. The pay grade was based on the current rank system, which was broken down into firefighter, senior fire sergeant, fire sergeant, fire lieutenant, and a position higher than fire lieutenant. In addition, we measured drinking habits using the Alcohol Use Disorders Identification Test (AUDIT) questionnaire [20]. Anyone who had 8 points or more in the AUDIT questionnaire was defined as a problematic heavy drinker [21]. Anyone who did not smoke for a week before the survey or who did not smoke at all was defined as a non-smoker.

The job stress factors were measured by the short form of the Korean Occupational Stress Scale(KOSS-26) [22]. The KOSS-26 inquires about the physical environment, job demands, insufficient job control, interpersonal conflict, job insecurity, organizational system, lack of reward, and occupational climate. The score of each sub-area of job stress survey was converted into units 100 point scale. In this study, the high-risk group was defined as higher than the 75th percentile, and the low-risk group as lower than the 75th percentile in each sub-class of job stress. The depression factors were measured by the Center for Epidemiologic Studies-Depression Scale (CES-D). Anyone who had 21 points or more on the CES-D questionnaire was defined as depressed [23]. Work-related musculoskeletal disorders were measured by the Korean NIOSH Symptom Survey [24] which consists of questions regarding the presence of work-related symptoms in any of 6 body regions (neck, shoulders, arms, hands/wrists, lower back, and legs/feet) in the prior year, and symptom severity (frequency, duration, and intensity). The frequency of the symptoms was rated on a 5-point scale ranging from 1 (every 6 months) to 5 (daily). The duration of the symptoms was rated on a 5-point scale ranging from 1 (less than 1 day) to 5 (6 months or greater). The intensity of the symptoms was rated on a 5-point scale ranging from 1 (mild pain) to 5 (very severe pain). WMSD cases were defined as symptoms that had occurred at least once a month or lasted at least one week in the past year and with at least moderate pain intensity [25].

### Statistical methods

The reliability coefficient of the job stress questionnaire was 0.799 and that of the CES-D was 0.875. The chi-squared test was used for the general characteristics of the firefighters and job stress. Uni-variate and multivariate logistic regression analysis were used to look for a correlation between general characteristics and job stress, and the occurrence of WMSD. Uni-variate logistic regression analysis was performed with each job stress subtype as an independent variable (Model I). Multivariate logistic regression analysis was performed with age, tenure, exercise, smoking, drinking, marital status, job title and job class as adjusted factors (Model II). Model III added depression to Model II as an adjusted factor. Statistical analyses were performed using the SPSS 17.0 for Windows software package (SPSS, Chicago, IL). All reported *p* values were two-tailed and those with results < 0.05 were considered to be statistically significant.

## Results

### General characteristics

The prevalence of WMSD was 11.0% (2,362 persons). The age of non-WMSD firefighters (39.6 ± 7.4) was statistically higher than the WMSD firefighters (39.0 ± 6.6). Non-WMSD firefighter work periods were statistically higher than those of the WMSD group. Statistical differences in marriage, education, problem drinking, exercise, main job, job class, and depression were observed between the two groups. The depression group was 17.7% (3,807 persons) (Table 1).

**Table 1:** General characteristics of subjects according to musculoskeletal symptoms

**General characteristics**		**Symptom (+)**		**Symptom (-)**		**p-value**
		**n = 2,362**		**n = 19,104**		
		**Mean**	**SD**	**Mean**	**SD**	
Age (years)		39.0	6.6	39.6	7.4	<0.01
Tenure (years)		11.1	6.7	11.7	7.5	<0.01
		n	%	N	%	
Marriage	Married	2,003	11.2	15,807	88.8	<0.01
	Single	359	9.8	3,228	90.2	
Education (years)	<16	894	10.9	7,295	89.1	<0.01
	≧16	1,468	11.1	11,809	89.9	
Problem drinking	Yes	881	10.1	7,875	89.9	0.02
	No	1,481	11.7	11,229	88.3	
Exercise	Yes	2,103	10.9	17,137	89.1	<0.01
	No	259	11.6	1,967	88.4	
Smoking	Yes	1,000	11.6	7,612	88.4	<0.01
	No	1,362	10.6	11,492	89.4	
Main job	Firefighting	1,113	10.5	9,476	89.5	<0.01
	Rescue	597	16.9	2,932	83.1	
	Emergency medical service	254	12.9	1,718	87.1	
	Others	398	7.4	4,978	92.6	
Job class	Firefighter	517	10.7	4,334	89.3	<0.01
	Senior fire sergeant	890	12.8	6,085	87.2	
	Fire sergeant	727	11.5	5,588	88.5	
	Fire lieutenant or higher	228	6.9	3,077	93.1	
Depression(CES-D) ^*^	< 21	1,608	16.0	16,051	84.0	<0.01
	≧ 21	754	31.9	3,053	68.1	

^*^: Center for Epidemiologic Studies-Depression Scale (CES-D).

### The ratio of complaints of WMSD

The most common pain site was the back with 1,294 (6.0%) complaints, followed by the neck with 724 (3.4%) complaints. There were fewer complaints about the foot, shoulder, hand, and arm (Table 2).

**Table 2:** Musculoskeletal pain locations in study subjects

**Region**	n^*^	**%**
Shoulder	462	2.2
Neck	724	3.4
Lumbar	1,294	6.0
Hand	225	1.0
Foot	714	3.3
Arm	162	0.8

^*^multiple choice.

### Logistic regression analysis of WMSDs according to job stress adjusted for the psychosocial component

Among those with job stress, only insufficient job control did not show statistical significance (Table 3). By adjusting the general characteristics, we found that physical environment, at odds ratio of 2.31 (95% CI, 2.03 - 2.62); job demand, at 1.55 (95% CI, 1.38 - 1.74); interpersonal conflict, at 1.15 (95% CI, 1.03 - 1.29); job insecurity, at 1.13 (95% CI, 1.02 - 1.25); organizational system, at 1.24 (95% CI, 1.10 - 1.38), and occupational climate, at 1.33 (95% CI, 1.19 to 1.49) were statistically significant (Model II). Adjusted for depression and general characteristics in the multi-variate analysis produced 2.22 (95% CI,1.96 - 2.53) for the physical environment; 1.52 (95% CI,1.35 - 1.70) for job demand; 1.14 (95% CI,1.01 - 1.28) for job insecurity; 1.37 (95% CI,1.21 - 1.58) for organizational system; 1.15 (95% CI,1.01 - 1.31) for lack of reward; 1.24 (95% CI,1.11 - 1.40) for occupational climate (Model III) (Table 4).

**Table 3:** Relationship between job stress and musculoskeletal symptom

			**Symptom(+)**	
	**Total(n)**	**n**	**Frequency(%)**	**p-value**
Physical environment				<0.001
High risk	10,825	1,700	15.70	
Low risk	10,641	662	6.22	
Job demand				<0.001
High risk	8,822	1,444	16.37	
Low risk	12,644	918	7.26	
Insufficient job control				0.027
High risk	9,048	1,046	11.56	
Low risk	12,418	1,316	10.60	
Interpersonal conflict				<0.001
High risk	8,529	1,137	13.33	
Low risk	12,937	1,225	9.47	
Job insecurity				<0.001
High risk	5,644	894	15.84	
Low risk	15,822	1,468	9.28	
Organizational system				<0.001
High risk	6,807	1,097	16.12	
Low risk	14,659	1,265	8.63	
Lack of reward				<0.001
High risk	5,683	897	15.78	
Low risk	15,783	1,465	9.28	
Occupational climate				<0.001
High risk	6,265	1,022	16.31	
Low risk	15,201	1,340	8.82	
Total job stress				<0.001
High risk	5,407	1,280	23.67	
Low risk	16,059	1,082		

**Table 4:** Adjusted odds ratio of musculoskeletal symptoms according to psychosocial factors

**Job stress**	**Model I**		**Model II**		**Model III**	
	Unadjusted OR*		**Adjusted OR**^**†**^		**Adjusted OR**^**‡**^	
	**O.R.**	**95% CI**	**O.R.**	**95% CI**	**O.R.**	**95% CI**
Physical Environment						
Low risk		1.0		1.0		1.0
High risk	2.37	2.14 - 2.63	2.31	2.03 - 2.62	2.22	1.96 - 2.53
Job demand						
Low risk		1.0		1.0		1.0
High risk	2.50	2.29 - 2.73	1.55	1.38 - 1.74	1.52	1.35 - 1.70
Insufficient job control						
Low risk		1.0		1.0		1.0
High risk	1.13	1.02 - 1.25	0.98	0.88 - 1.01	0.99	0.88 - 1.10
Interpersonal conflict						
Low risk		1.0		1.0		1.0
High risk	1.60	1.44 - 1.70	1.15	1.03 - 1.29	1.11	0.99 - 1.24
Job insecurity						
Low risk		1.0		1.0		1.0
High risk	1.90	1.71 - 2.11	1.13	1.02 - 1.25	1.14	1.01 - 1.28
Organizational system						
Low risk		1.0		1.0		1.0
High risk	2.22	2.01 - 2.47	1.24	1.10 - 1.38	1.37	1.21 - 1.56
Lack of reward						
Low risk		1.0		1.0		1.0
High risk	1.92	1.73 - 2.13	1.23	1.08 - 1.39	1.15	1.01 - 1.31
Occupational climate						
Low risk		1.0		1.0		1.0
High risk	2.16	1.94 - 2.39	1.33	1.19 - 1.49	1.24	1.11 - 1.40

^*^: Model I:Odds ratio (O.R). confidence interval (CI).

^†^: Model II: adjusted by age, tenure, exercise, smoking, drinking, marital status, job title, job class.

^‡^: Model III: depression added at model II as a adjusted factor.

## Discussion

As with the previous study [25], back pain was the most common WMSD in firefighters. This study shows that job stress may have an association with WMSDs in firefighters. Among the job stress subgroup, physical environment, job demand, job insecurity, lack of reward, and occupational climate were associated with WMSDs.

Because fire-fighting is urgent and unpredictable, it is known to generate extreme psychological and physical burdens in the line of duty [26]. When rescuing victims and extinguishing fires, WMSDs can arise due to the unbalanced body positions resulting from the use of heavy fire protection equipment [19]. Back pain is caused by various factors including poor posture, degenerative changes, and psychological states. The principal factor is a bio-mechanical factor because of mechanical stress, particularly heavy weight-handling work. Excessive force is put on the lower back when firefighters use heavy equipment [25]. Work situations that cause back pain for firefighters include operating a charged hose inside a building (odds ratio (OR) = 3.26), climbing ladders (OR = 3.18), breaking windows (OR = 4.45), cutting structures (OR = 6.47), looking for hidden fires (OR = 4.32), and lifting objects ≥18 kg (OR = 3.07) [27]. The risk gradually increased as the fire fighter moved away from the firehouse (OR = 0.10) and closer to the site of the fire (OR = 3.91) [27].

WMSD is a multi-factorial disorder related to various demographic and general features and work-related characteristics [28,29]. Social-demographic features, work-related characteristics, health-related behaviors, and job stress can all affect social psychological health levels [30]. Old age, unemployment, past musculoskeletal pain, and shift work are factors that extend the symptoms [31]. There is a hypothesis that muscle strain increases if job-related psychological burdens increase due to job stress, and job stress increases awareness of musculoskeletal symptoms or reduces the capability to manage the symptoms [25,32,33]. Unemployment experience, monthly income, lack of sleep, and drinking status acted as major cause of stress [34]. Job insecurity, interpersonal conflicts, the physical environment, and organization systems had negative impacts on the level of mental stress of firefighters [35]. The physical environment had a greater effect on field service workers than office workers [36]. The total job stress of firefighters was similar to that of general workers, but the physical environment factor was higher with firefighters than with general workers. Job insecurity and lack of rewards had somewhat less impact on firefighters than general workers [6]. As pointed out previously, firefighting is dangerous and requires best practices in handling bodily burdens. A lack of reward was associated with WMSDs and the same result has been observed in previous studies [25,37], and in office workers [38].

However, insufficient job control does not show as much correlation with WMSDs as a consistent lack of reward [25]. In the present study, a correlation could not be found. The organizational system score evaluates the organization of political tactics, operating systems, organizational resources, and reasonable communication within the community [22]. In subway workers, the organization system score was statistically significantly higher (bad) than in firefighters [36]. The authors believe that South Korea’s authoritarian and vertical organization system and lack of communication would tend to increase the development of WMSDs.

Depression is known to be related to job stress [39] and Kim *et al.*[40] have noted that the job insecurity and depression level is high in emotional labor workers. Depression is a major factor of absence and labor injuries [41], and it is known as an independent precursor to neck and lumbar pain [42]. In firefighters, a change in workload, discord at work, role conflicts, and low pride had links to depression [34]. In a previous study, 33% of the firefighters had symptoms of depression, and firefighters who worked at the fire department had symptoms of depression 2.1 times more frequently than those who worked in the business administration department [43]. In our study, WMSD firefighters had 1.86 times more symptoms of depression then those who had not (95%CI:1.65-2.09).

In a previous report, the distribution of firefighter diseases included circulatory system diseases (26%), musculoskeletal abnormalities (25%), and psychiatric problems (16%) [44]. The most common causes for retirement were musculoskeletal disorders (25%), nervous system and sense organ disorders (24%), and alimentary system problems (13%) [44]. The prevalence of WMSDs (11%) in male Korean firefighters was lower than in the previous study in Korea. However, musculoskeletal symptoms accounted for a significant portion of the firefighter’s disabilities. Therefore the management of WMSDs are also known to be necessary for prevention in medical and engineering management and psychosocial management [45].

The limitations of this study were as follows. First, there was no physical examination or ergonomic evaluation for defining WMSDs. The personal protective equipment (self-contained breathing apparatus (SCBA), protective cloth, etc.) of a firefighter weighs just under 25 kg, with the SCBA at about 15 kg [18]. We could not rule out the effect of the heavy equipment and ergonomic factor and they worked as confounding factors in our study.

Another limitation was study design. This study was a cross-sectional study, so further studies are necessary to determine the cause-effect relationship between the studied factors. In spite of these limitations, the study did evaluate enough people to represent all of the male firefighters in Korea.

## Conclusion

Our result suggests that job stress may act in firefighters as a factor of WMSD development. Work to reduce job stress and a care system to mange it are needed for firefighters.

## Consent

This research was approved by the Institutional Review Board at Occupational Safety and Health Research Institute. Written informed consent was obtained from each participant prior to their involvement in the study.

## Competing interests

The authors declare that they have no competing interests.

## Authors’ contributions

Kim K-S conceived and designed the study. All authors developed research model, especially Kim MG analyzed the statisitcs and wrote the manuscript. All authors read and approved the final manuscript.
